# A Novel Ear Impression-Taking Method Using Structured Light Imaging and Machine Learning: A Pilot Proof of Concept Study with Patients’ Feedback on Prototype

**DOI:** 10.3390/jcm13051214

**Published:** 2024-02-21

**Authors:** Kenneth Wei De Chua, Hazel Kai Hui Yeo, Charmaine Kai Ling Tan, Jose C. Martinez, Zhi Hwee Goh, Stylianos Dritsas, Robert E. Simpson

**Affiliations:** 1 Department of Otorhinolaryngology-Head and Neck Surgery, Allied Health, Audiology, Changi General Hospital, Singapore 529889, Singapore; 2 Department of Electronic, Electrical and Systems Engineering, Singapore University of Technology and Design (SUTD), 8 Somapah Road, Singapore 487372, Singapore; 3 Department of Architecture and Sustainable Design, Singapore University of Technology and Design (SUTD), 8 Somapah Road, Singapore 487372, Singapore

**Keywords:** innovation, ear, ear canal, three-dimensional scanning, proof of concept study

## Abstract

Introduction: Taking an ear impression is a minimally invasive procedure. A review of existing literature suggests that contactless methods of scanning the ear have not been developed. We proposed to establish a correlation between external ear features with the ear canal and with this proof of concept to develop a prototype and an algorithm for capturing and predicting ear canal information. Methods: We developed a novel prototype using structured light imaging to capture external images of the ear. Using a large database of existing ear impression images obtained by traditional methods, correlation analyses were carried out and established. A deep neural network was devised to build a predictive algorithm. Patients undergoing hearing aid evaluation undertook both methods of ear impression-taking. We evaluated their subjective feedback and determined if there was a close enough objective match between the images obtained from the impression techniques. Results: A prototype was developed and deployed for trial, and most participants were comfortable with this novel method of ear impression-taking. Partial matching of the ear canal could be obtained from the images taken, and the predictive algorithm applied for a few sample images was within good standard of error with proof of concept established. Discussion: Further studies are warranted to strengthen the predictive capabilities of the algorithm and determine optimal prototype imaging positions so that sufficient ear canal information can be obtained for three-dimensional printing. Ear impression-taking may then have the potential to be automated, with the possibility of same-day three-dimensional printing of the earmold to provide timely access.

## 1. Introduction

Hearing aid amplification sometimes requires customized earmolds to be made to assist device fixation in the ear canal. The earmold also provides a better acoustic seal to minimize auditory feedback. This mold construction requires acquiring an impression of the ear canal (ear impression). Although this procedure is generally safe, there are known complications, such as inflammatory reaction, bruising, and eardrum perforation, reported [1]. When an ear impression needs to be taken, it requires a trained and skillful audiologist. In Singapore, ear impressions are generally taken by audiologists, whether in public or private practice. This standard procedure, however, is time-consuming and slightly invasive. From otoscopy inspection to inserting the foam block (Oto-Block) to stop the flow of the silicone mixture to finally removing the ear impression, takes approximately 10–15 min [2]. This takes up significant consultation time, which could be better utilized in other clinical procedures to improve patients’ overall experience with hearing aids. Ear impression-taking may also be uncomfortable, and at times there may be ear canal bruising and bleeding after the procedure. Some patients with anxiety also do not tolerate this procedure well and may have to come back for repeated measures. Impression-taking is also contraindicated when there is significant ear wax occlusion, active ear infections, or post-surgical ear cavities. In some cases, surgical removal of the ear impression as a foreign body is required and has been reported in many studies [3,4,5,6]. The total direct and indirect cost of ear impression-taking also amounts to about SGD 40,000 per year (unpublished local data). Hence, there is clearly an unmet need to innovate, to make impression-taking non-invasive, quick, and low-cost.

Our objective is to develop, prototype, and trial an efficient method to create customized earmolds for hearing aids. The current method of obtaining an ear impression is to mix a silicone base and catalyst together and syringe the mixture into the patient’s ears. The proposed solution is to use optical imaging to capture data points of the external ear and use machine learning (ML) to predict the 3D image of the ear impression. This ML algorithm will be developed and refined using a large database of existing 3D ear impression images obtained previously via traditional techniques. Although there are existing 3D ear scanning devices on the market, contact with the ears still needs to be made, leaving these available systems still minimally invasive. Otoscan, for example, requires a probe to be inserted into the ear canal (invasive) and is relatively expensive. Hence, we reviewed the literature for existing 3D scanning technology with the goal of developing a prototype for accurately measuring portions of the ear canal directly without physical contact. For a detailed literature review of existing 3D scanning methods, please refer to the previous study [7].

Three contactless 3D scanning technologies were eventually identified for comparison [7], namely photogrammetry, structured light, and laser-line scanning techniques. Using a 3D-printed control ear, all three scanning techniques were evaluated, and a scoring matrix was used [7]. While photogrammetry was suggested to be easy to use, its resolution was poorer than the structured light and laser-line techniques. Between the latter two, structured light emerged as a clear winner in terms of resolution and time involved from scanning to the post-processing of images [7]. A prototype was developed by the engineering team, which enabled audiologists to take digital impressions of the ear instead of the usual physical silicone ear impression. This non-invasive method reduces clinical risk and discomfort to the patient and reduces carbon dioxide emissions from ground transportation between the clinic and hearing aid manufacturers when ear impressions are couriered. To our knowledge, there are no studies leveraging big data and artificial intelligence to model the geometry of patients’ ears using a direct scan of the outer-ear pinna and an ML-built model of the ear canal. This novel approach is expected to profoundly change the methods used by audiologists, improve the experience of patients, and deliver tangible cost savings.

## 2. Materials and Methods

### 2.1. Prototype Development, Phase I

The ear scanner prototype is easy to use with the rail and carriage concept, ensuring consistency in the scanning path to obtain a quality ear impression. The function of the prototype for ear impression application is to take a digital ear impression without the need to insert material into the patient’s ear or have physical contact with the patient. The prototype will be able to generate a partial 3D mesh model of the patient’s ear, which the primary user can export or download for further processing.

The primary users of the device for ear impression applications are audiologists and experts in their medical field who are not expected to be in the technology domain. The secondary users of the device for ear impression application are the patients.

**Figure 1:**
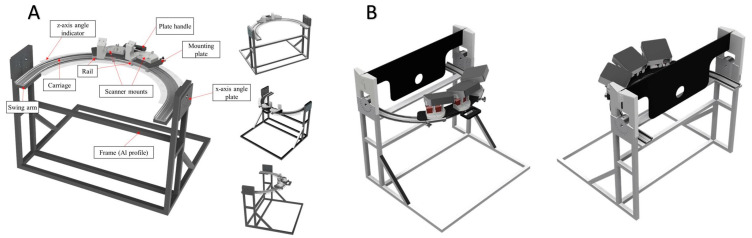
(**A**): A close-up sketch of the ear scanner prototype, (**B**): Adjustable height with acrylic frame showing a circular hole where the ear is placed.

A sketch of the prototype is seen in Figure 1.

The ear scanner prototype consisted of a frame-to-carriage adjoining bracket and an L-shaped arm bracket, to account for the scanner’s lens relative to the patient’s ear. The size of the prototype frame was also designed to be placed on a tabletop. The ear scanner is designed with a rail–carriage system to allow rotation of the scanner along the z-axis and an angle plate to allow rotation of the scanner along the x-axis relative to the scanned object, a patient’s ear. A lubrication-free rail–carriage system with a curved profile was acquired from Igus, a German company, which has a curved rail–carriage system with patented bearing technology. The bearing technology utilized on Igus’ curved rail–carriage system operates without any lubrication requirement, so the risk of splashed grease or oil exposed in the clinic was reduced to zero. The angle plate and main frame of the prototype are assembled with a 20 series aluminum profile.

As not every patient’s ear will always be aligned to the center of the scanner due to patients’ height differences, the prototype is designed to be placed on a height-adjustable table. This mechanism will allow the accommodation of patients of various heights. A black acrylic plate and a 3D-printed bracket attached to the top of the prototype provide a guide into which the patient must slot their ear while leaning against the plate. The black acrylic plate doubles as an eye protector to protect the patient’s eyes from the projector’s light source while taking the ear impression.

### 2.2. 6500 3D Ear Impressions Obtained for Machine Learning, Phase II

**Figure 2:**
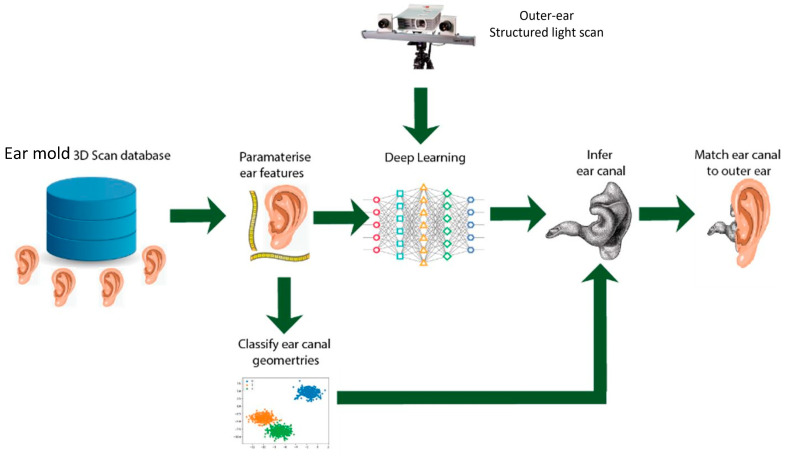
Schematic showing structured light scanning with machine learning on ear impression images to predict and classify ear canal shapes.

Although the prototype captured most parts of the outer ear (pinna) as well as parts of the ear canal, some predictive algorithm is necessary to generate the predicted ear canal shape and length. From traditional physical ear impressions obtained over the past 3 years, we managed to retrieve approximately 6500 3D ear impression images for deep neural network training to establish the correlation between the outer parts of the ear and the ear canal. Our approach is to predict the dimensions of the ear canal using a deep neural network that has been trained on a database containing 6500 ear 3D images, to predict the dimensions of the ear canal using a directly scanned model of the patient’s outer ear, using our developed prototype mentioned above. The overall scheme is depicted in Figure 2. To predict the ear canal shape from external imagery, we developed a convolutional neural network (CNN) supervised machine learning model. Using the scanned data sets already available or obtained, we trained the CNN on 3000 images by inhibiting the canal‘s shape and revealing only the external ear shape. Due to the laborious nature of data processing, we did not manage to train all 6500 images in the neural network, but the network trained in this pilot study was sufficient for a proof of concept. The experimental data from the 3000 images can be found in our recent study (unpublished data). Most of the anatomical information from the physical ear impressions used for training the neural network was derived from the ear canal. The data were limited about the external parts of the ears that could be obtained from the physical ear impressions. Hence, the challenge was in determining the start and end points of the ear canal. Without much information on the external ear, applying the algorithm eventually to prototype images of the external ear became a limitation in its prediction.

The CNN predicts the parameters of the ear canal model through stochastic gradient descent training against the known ground truth models. It will develop a non-linear regression model not only capable of predicting the presented data but also unseen shapes. Using approximately 70,000-point cloud data giving the coordinates of the points of the canal wall, an XYZ reference frame was created with each point cloud data yielding 12 to 36 elliptic cross sections. A lexicon for classifying ear canal shapes and sizes was also created from internal validation of 300 ear image samples to better categorize different shapes and sizes of ear canals [8]. Seven ear canal types were identified, and this laid the foundation for predicting ear canal shapes from incomplete scans, such as those obtained by the prototype.

### 2.3. Deployment of Prototype for Clinical Trial, Phase III

A total of 19 patients (38 ears) attending an audiologist for hearing aid evaluation (HAE), who required ear impression taking, were recruited. All the patients undertook both traditional ear impression-taking and novel structured light scanning in a randomized order. They were asked for their subjective feedback on the process including rating the comfort of both procedures on a Likert scale. To maintain consistency in the imaging technique, only one audiologist was responsible for using the prototype to scan patients’ ears. The time taken for physical ear impression-taking was also noted for comparison. This study was reviewed by SingHealth’s Centralized Institute Review Board (CIRB), and approval was given (2020/3140) prior to recruitment. Physical ear impressions via the traditional method were also taken from the same patient. The physical impressions were then scanned using our 3D scanner and exported for analyses vis-à-vis the prototype images. Objective comparisons were made to look at the degree of closeness and correlation between both types of ear impression images.

### 2.4. Brief Description of Structured Light Technique Using Novel Prototype

Structured light imaging is a process that captures the three-dimensional topography of a surface using specific patterns of light (often grids or horizontal bars). In our prototype, we projected parallel black and white stripes on the patient’s ear through an acrylic frame, with a circular hole for the ear placement. As the pattern illuminates the physical ear, it deforms and creates different depths of the ear, which are captured by the 2D cameras. The captured image of the deformed pattern is then compared to the original pattern, and the differences are used to calculate the depth and surface of the physical ear. This information is finally reconstructed into a 3D model. The patient will be seated comfortably on a chair and will put his ear through the ear hole located on a black acrylic frame, as seen in Figure 1b. The patient will be instructed to minimize movement, and the first image will be captured at 0-degree azimuth on the X-axis. In incremental gains of +/−20 degrees, up to +60- and −60-degree azimuth, another six images will be obtained. As the prototype was mounted on an adjustable table, we were able to adjust the prototype to the patient’s height. A z-axis rotator bolt was then used to angle the project in the vertical plane at + and −15 from Earth’s vertical axis. In each vertical plane, the same 7 images were obtained at 0-degree azimuth up to +/−60-degree azimuth. A total of 7 s is required for capturing one image of the ear from one angle. We captured a total of 21 images per ear, and it took approximately 300 s (5 min) to capture 42 images from both ears, per patient.

## 3. Results

### 3.1. Patients’ Subjective Feedback

**Figure 3:**
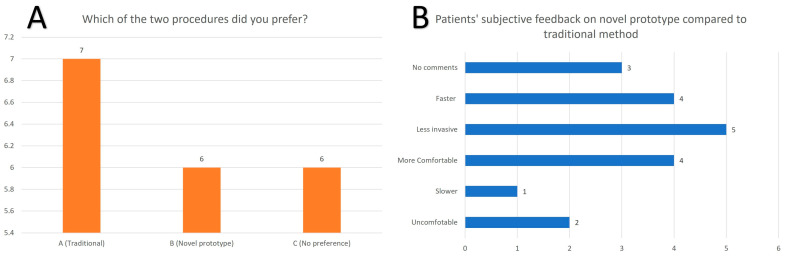
(**A**): Patients ‘preference for type of ear impression method; (**B**): Summary of patients ‘subjective feedback on novel prototype method.

There were no adverse or serious adverse events arising from the use of the prototype on patients. All but one patient tolerated the procedure well during the image-capturing process. Of the 19 patients, 37% (7/19) rated the traditional ear impression technique more favorably (Figure 3a), while 31.5% (6/19) preferred the novel prototype or had no preference for either method. The summary of the patients’ feedback can be seen in Figure 3b. The time taken on average for a physical ear impression was approximately ten minutes, which seems to be longer than the prototype imaging. However, this does not take into account the total time taken for post-editing of the prototype images, which was not necessary in the traditional method. One patient suggested that the novel prototype imaging was taking too long, but the patient was also noted to be fidgeting, which resulted in several repeated measures.

### 3.2. Objective Comparative Analyses of 8 Samples of Prototype Images with Actual Physical Ear Impressions

**Figure 4:**
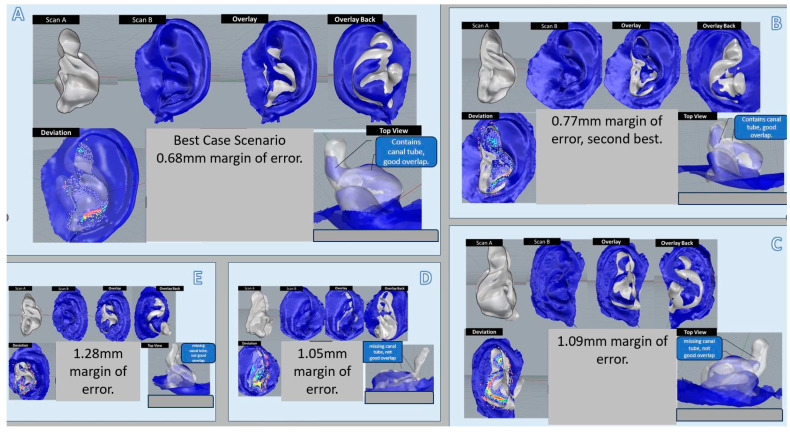
Comparative analyses between physical ear scans and digital ear impressions. (**A**,**B**): Best correlation between structured light image and traditional image. (**C**–**E**): Moderate to poor correlation.

Eight images of the ear taken by the prototype were randomly selected. These images were compared with actual physical impressions taken from the same ear. We looked at registration error, which measures point distances of 4000 random points. The comparison analyses were poor for three of those images, especially for predicting ear canal information past the second bend, due to noise artifacts from the prototype imaging. There was, however, a good match between both the prototype image and the physical ear impression. Looking at the five prototype images (Figure 4A–E) for comparison, both projection distances after alignment and scaling factor errors were within an average of a 1 mm margin of error (ranging from 0.68 mm mean error in the best case to 1.28 mm mean error). The rest of the images were within a 2.5 mm margin of error and considered to be a poor prediction. The predictive algorithm, when applied to the best prototype image obtained, was close to a 100% match and correlated well with the physical ear impression of the same ear. Handedness was also successfully predicted by the algorithm on physical ear impressions. However, when the algorithm was applied to prototype images, only 50% of the images predicted handedness correctly. We sent the most ideal prototype image with predicted ear canal parts to a local hearing aid manufacturer to attempt 3D printing of the earmold. However, after laboratory expert analyses and processing of the image, it was determined that there was still insufficient ear canal information for a gold-standard printing of hearing aid earmolds.

## 4. Discussion

This novel ear impression method will potentially allow us to take digital ear impressions even with contraindications of the ears. There is also a potential for cost savings, as ear impression materials with courier costs are expensive. The estimated cost per ear impression is SGD 4.30, and on average ten impressions are taken in a day. This amounts to about SGD 43 a day and SGD 251 a week, including a two-way courier cost. Over a year, this may result in savings of SGD 13,052. Furthermore, there are also indirect and intangible costs due to extra time taken in the traditional physical ear impression method, which may be an opportunity cost of at least SGD 105 per day and approximately SGD 27,300 in a year. Although there is still a substantial amount of missing information and prediction capabilities are not high yet—especially for the canal information past the second bend—most hearing aid molds will not require such a deep canal length, unless it is for a custom in-the-canal or completely-in-canal hearing aids [9]. Patients found the structured light technique to be of greater comfort and convenience without any probing or poking of the ear canal. Turnaround time may also be faster with improvements in imaging techniques, without any downtime waiting for the ear impression materials to harden. There is no risk of canal bruising, bleeding, or the ear impression trapped as a foreign body. With further iteration of the design, we hope to make the prototype smaller and more portable. The main difference in the images obtained between the physical ear impression and the prototype is the amount of ear canal information. As the physical impression materials fill up the entire ear canal, predicting the shape of the ear canal with physical impression data is easy. However, with structured light imaging, we were unable to get as much information about the ear canal compared to the traditional method. Hence, the neural network algorithm was not as sensitive as the training was based on the available ear canal information of the 3000 images. Hence, prospective studies should also evaluate the optimal scanning angles on the X and Z axis for the greatest accuracy in obtaining ear canal information. However, the main limitation of this prototype in its current form is that it is very sensitive to noise artifacts from movements. Hence, future iterations and improvements to this prototype should include modifications to minimize head and body movements.

Our next step forward will be to curate and expand the dataset of physical ear impression images and ensure quality control in the images obtained. Of the 6500 images obtained in this study, many were of poorer quality, as the quality of physical ear impressions varies based on the skill of the audiologists. Furthermore, there was considerable digital processing of the 3D-scanned images without access to the raw data. To minimize variability in quality, physical ear impressions should be taken in future studies by one or two highly skilled audiologists, who can ensure the quality of the data obtained. Ear impressions should also be directly scanned with 3D scanners, and raw data should be used for further deep neural network training. This can be conducted by blinding the 3D images to predict the ear canal shape from pictures taken from the same ear. Many of the prototype images were contaminated with noise artifacts. This may be due to physical movements during the imaging process. Further design improvements of the prototype, including ergonomics of the seat, are important considerations to minimize patient movements. A comfortable head-restrainer or a reclining chair may be necessary to relax the patients and put them in the calmest state during prototype imaging. Determining the optimal x- and z-axis angles for imaging is also crucial, and this may be achieved through trial-and-error analyses. As far as we know, there are no existing studies on contactless scanning methods for taking an ear impression. The existing literature has suggested the use of Otoscan, whose main disadvantage is still the use of a semi-invasive probe that must be inserted into the ear canal to a certain depth to complete a 3D scan of the ear canal. Although this method provides better accuracy in providing ear canal information compared to our prototype, direct comparisons cannot be made, as we are looking at a contactless novel and innovative method of ear impression-taking. Therefore, we believe that future improvements and iterations of the prototype with refinements in machine learning will result in a safer and smarter method of ear impression-taking.

## 5. Conclusions

A pilot prototype was developed, and a correlation between the external ear features and the ear canal was established. Although this was not sufficient to build a highly predictive algorithm, the proof of concept was still valid, and some patients appreciated the non-invasive method of ear impression-taking. With continuous work on the prototype to capture more details of the ear canal and further training of the neural network, we may be able to produce a better algorithm. Furthermore, a trial-and-error analysis of the optimal angles required to generate most ear canal information will further fine-tune this process. If the prototype can be miniaturized and commercialized, this may allow for tele-audiology services, such as hearing aid dispensing and fitting on the same day, as ear impression-taking can potentially be automated and combined with 3D printing [10]. This will improve accessibility to hearing health as we transit to a population health landscape.

## Data Availability

Available upon request.
